# Postural Balance, Hearing Loss, and Cognitive Executive Function in the Elderly: Are They Related?

**DOI:** 10.3390/audiolres16050125

**Published:** 2026-08-26

**Authors:** Carla Rodríguez-Zanetti, Janaina Patricio de Lima, Manuel Manrique, Diego Calavia, Raquel Manrique-Huarte

**Affiliations:** Department of Otorhinolaryngology, Clínica Universidad de Navarra, 31008 Pamplona, Spain; janaina.patricio@hc.fm.usp.br (J.P.d.L.); mmanrique@unav.es (M.M.); dcalaviag@unav.es (D.C.); rmanrique@unav.es (R.M.-H.)

**Keywords:** hearing loss, postural imbalance, cognition, adults, ageing

## Abstract

**Background**: There might be a significant association between hearing loss (HL) and cognitive performance or postural imbalance, but how they relate to each other remains unknown. The aim of this study is to gain an understanding of these relationships. **Methods**: An observational prospective study with subjects ≥ 55 years old divided into groups: normal hearing (group A), untreated HL > 20 decibel hearing level (dBHL) (group B), and patients with treated HL > 20 dBHL (group C). At the initial visit and follow-up, participants underwent tests and questionnaires evaluating hearing, balance, and cognition. Statistical analyses—including relative risk, Spearman’s Rho correlation, interaction, and adjusted models—were performed using Stata 17; a *p*-value < 0.05 was considered statistically significant. **Results**: In total, 714 subjects were initially recruited: 210 in group A, 302 in group B, and 202 in group C. A mean follow-up time of 3.6 years was completed by 101 individuals. Compared with the normal-hearing group, hearing loss increased the probability of unsteadiness by 1.4-fold in group B and 4.1-fold in group C. Poorer performance on the Timed Up and Go (TUG) test was predicted by lower scores on both cognitive executive function tests, with correlations of −0.4 and 0.34 (*p* < 0.001). Regarding confounding factors, statistically significant differences were found for age and education in cognitive performance; age in the TUG results; and sex, cardiovascular risk factors (CVRF), and education in HL. **Conclusions**: Postural imbalance and cognitive executive function are related to HL. However, there is no evidence linking dizziness to cognitive executive function, although an association with TUG results was found. Therefore, HL is correlated with both balance and cognition, and cognition is also related to balance.

## 1. Introduction

Age-related hearing loss (ARHL) is a gradual hearing loss that most people suffer as they age [1]. The term encompasses all conditions that lead to hearing loss in elderly people. There are three classic types of this disorder—sensory, strial, and neural—which can occur alone or in combination [2]. The most common pattern is bilateral, progressive sensorineural hearing loss that particularly affects high frequencies.

According to Roth et al. [3], roughly 30% of men and 20% of women in Europe have hearing loss of 30 dBHL or more by 70 years of age, and 55% of men and 45% of women have the same by 80 years of age. Age-related hearing loss is a prevalent and clinically relevant communication disorder that involves alterations to the cochlea and central auditory pathway [4], and it is characterized by reduced hearing sensitivity and speech understanding in noisy environments, slowed central processing of acoustic information, and impaired sound localization [2]. It significantly lowers the quality of life in the elderly and is linked to other “non-auditory features”, such as balance issues, falls, social isolation, depression, and cognitive impairment [5]. According to a report on dementia prevention, when protective and detrimental factors are considered, hearing aid use has been identified as the largest modifiable factor for preventing cognitive deterioration [6]. Given these findings, it is crucial to understand how ARHL affects non-auditory domains.

Alteration of the vestibule or vestibular pathway is highly prevalent in older adults, with nearly 50% of individuals over 60 years of age demonstrating some form of physiological vestibular loss [7,8]. Age-related vestibular loss (ARVL), defined as a loss of vestibular function, is characterized by unsteadiness, dizziness, and/or vertigo, as well as disturbances in gaze and postural stability or balance impairment [9]. This entity has also been associated with an increased rate and likelihood of cognitive decline in older adults, resulting in increased risk of falling [10,11,12].

Several mechanisms could explain an association between hearing loss and vestibular loss, but whether this reflects a concomitant dysfunction, causal relationship, or other mechanism that may affect both simultaneously, such as cognitive decline or ageing itself, is still under investigation. The mechanisms may appear in isolation or together, with considerable negative consequences for quality of life, and data on how these conditions affect each other in this population remain insufficient. In our previous study, we established a relationship between hearing levels and results on balance tests—the Timed Up and Go (TUG) test and the Dizziness Handicap Index (DHI)—showing that scores on these tests worsened with increasing hearing impairment [13], underscoring the importance of studying these associations.

Executive function is among the most advanced cognitive activities of the human brain and is responsible for controlling and coordinating various specific cognitive processes. Studies show that executive function is not a cognitive system with a single structure but rather involves multidimensional structures, including inhibitory control, attentional control, working memory, and cognitive flexibility, all of which are affected by ageing [14].

Furthermore, falls and issues with hearing and balance are prevalent among the elderly. Whether hearing loss is a modifiable risk factor for preventing falls in older adults remains an open question. Identifying modifiable risk factors for falls in older people is critical for public health [15]. Although hearing is not typically considered a risk factor for falls [16,17], audiometric hearing loss has been associated with incident falls; indeed, Lin and Ferrucci [18] found 1.4-fold (95% CI, 1.3–1.5) increased odds of an individual reporting a fall over the previous 12 months.

It is also important to mention that common etiological or risk factors could underlie all three conditions in the elderly, such as genetic factors, environmental factors, ageing itself, and chronic diseases, among others [14]; these should also be studied.

The aim of this study was to investigate the association between hearing loss, postural balance, and cognitive decline in the elderly and identify factors that may be associated with the development of these conditions in this population.

## 2. Materials and Methods

This study is part of an ongoing project entitled “Hearing and balance in healthy ageing.” The project was approved by the Clinical Research Ethics Committee of the University of Navarra Clinic (file number 2017.174). Written informed consent was obtained from participants in the study.

Population

Subjects were recruited at the Otorhinolaryngology Department of the Clínica Universidad de Navarra and through an advertisement in a local newspaper requesting participation. Participants demonstrated fluency in Spanish for clinical assessment, expressed willingness to engage in and adhere to the study protocol, and had the capacity to independently consent to participation. Three subgroups of the population were studied for this purpose, with a mean follow-up of 3.6 years. As this is an ongoing study, many other participants are still awaiting follow-up; at the time of recruitment, 714 participants had been enrolled, whereas at the time of the analyses for this study, 101 participants had completed the follow-up visit. The inclusion criteria for each of these groups are described in the following section.

Inclusion Criteria:

- Group A inclusion criteria required individuals aged 55 years or older, with normal hearing in both ears, defined as a pure-tone average (PTA) of 0–20 dBHL across 500, 1000, 2000, and 4000 Hz.
- Group B inclusion criteria required individuals aged 55 years or older diagnosed with hearing loss > 20 dBHL who had not undergone treatment.
- Group C inclusion criteria required individuals aged 55 years or older diagnosed with unilateral or bilateral hearing loss > 20 dBHL who had received treatment with hearing aids (HAs), active middle ear implants (AMEIs), bone conduction implants (BCIs), or cochlear implants (CIs).

Exclusion Criteria: Included individuals who were significantly or severely dependent on others or considered frail; those incapable of personally providing consent or unable to independently complete self-assessment questionnaires; those with ossification or other cochlear anomalies impeding full electrode insertion in the case of cochlear implants (CIs); those with retrocochlear or central causes of hearing impairment; those with substantial comorbidities hindering study participation (e.g., blindness, immobility, severe aphasia); or those with clinical treatment failure related to chronic depression, dementia, or cognitive disorders. Conditions such as sudden, idiopathic, or post-traumatic hearing loss were not excluded, as these result in more severe hearing impairment than physiological presbycusis and will have a greater overall impact within the concept of ageing.

Study Measures:

The anamnesis, including medical history and the presence of symptoms such as vertigo and unsteadiness, as well as the physical examination, were first performed by an otorhinolaryngology specialist physician. All assessments were performed or administered by an accredited audiologist or audiology specialist technician experienced in audiological assessment.

- *Hearing assessment tools:* Pure-tone average (PTA) was used as the indicator of auditory thresholds in our sample. Bone-conduction assessment was performed only to classify the type of hearing loss (conductive, sensorineural, or mixed) according to clinical standards and was not used as a variable in any analysis.
  (a) Unassisted pure-tone air-conduction audiometry: This assessment was conducted in soundproof chambers using an Interacoustics audiometer, model AC40 (Diatec Diagnostics, Madrid, Spain). Unaided hearing thresholds, measured in decibel hearing level (dBHL) for pure-tone stimuli via air conduction, were assessed using TDH-39 headphones following standard clinical procedures. Frequencies ranging from 250 to 4000 Hz were evaluated (250, 500, 1000, 2000, and 4000 Hz), and a pure-tone average was calculated. This routine assessment typically takes around 15 min and involves both the clinician and the participant.
  (b) Unassisted pure-tone bone-conduction audiometry: This assessment was conducted in the same soundproof chambers using the Interacoustics AC40 audiometer (Diatec Diagnostics, Madrid, Spain). Unaided bone-conduction thresholds, measured in decibel hearing level (dBHL), were assessed using the bone vibrator positioned on the mastoid process of the tested ear, according to standard clinical procedures. Frequencies ranging from 500 to 4000 Hz were evaluated (500, 1000, 2000, and 4000 Hz). This routine assessment typically takes around 15 min and involves both the clinician and the participant.
- *Dizziness and balance assessment tools:* The TUG test was our main objective test to measure postural balance. The DHI reflects the self-perceived impact of dizziness and sometimes unsteadiness and is especially useful for measuring health-related quality of life. Finally, symptoms of vertigo and unsteadiness, although subjective, provide a useful means of assessing patients’ perception of their postural control.
  (a) Timed Up and Go Test: TUG evaluates mobility, fall risk, balance, and walking pattern [19]. It measures the time taken for a person to stand up from a standard armchair, walk 3 m (10 feet), turn around, walk back to the chair, and then sit down again. The test is performed with the patient wearing regular footwear, using their usual assistive devices if applicable, and walking at a comfortable and safe pace. The clinician uses a stopwatch to accurately time the complete manoeuvre, and the number of seconds taken is recorded. The test is performed at each follow-up assessment interval [20]. This test can be completed by the clinician with the candidate in approximately 3 min.
  (b) Dizziness Handicap Inventory (DHI): The DHI is a validated self-assessment questionnaire designed to evaluate the impact of self-perceived dizziness on a patient’s daily life, including functional, emotional, and physical domains. Several studies have demonstrated its utility in correlating with various measures of balance and gait performance. It is a 25-item inventory designed to evaluate the self-perceived handicapping effects of dizziness and unsteadiness [21]. Patients are asked to answer each question in relation to dizziness or unsteadiness problems experienced over the previous month. The questions are designed to capture functional (F), physical (P), and emotional (E) impacts on disability. Each item can be scored as follows: No = 0; Sometimes = 2; Yes = 4. The three domains—F (9 items, 36 points), E (9 items, 36 points), and P (7 items, 28 points)—sum to a total of 100 points.
  (c) Vertigo symptoms: During the anamnesis, patients were asked about the presence or absence of vertigo symptoms, and yes/no responses were recorded.
  (d) Unsteadiness symptoms: During the anamnesis, patients were asked about the presence or absence of unsteadiness symptoms, and yes/no responses were recorded.
- *Cognitive assessment tools:* TMT-B and DSST are objective tests that assess cognitive executive function and its changes over time.
  (a) Trail Making Test Part B (TMT-B): The Trail Making Test Part B [22] is a neurophysiological test assessing executive function requiring visual search, scanning, speed processing, and mental flexibility. TMT-B consists of 25 circles distributed over a sheet of paper. The circles include both numbers (1–13) and letters (A–L); the patient draws lines to connect the circles in an ascending pattern, alternating between the numbers and letters (i.e., 1-A-2-B-3-C, etc.). Patients are instructed to connect the circles as quickly as possible, without lifting the pen or pencil from the paper. The time taken to connect the “trail” is scored. If the patient makes an error, it is pointed out immediately, and the patient is allowed to correct it. The number of errors made are registered. The correction of errors is included in the completion time for the task. The test is stopped after 5 min. This form can be completed in around 5 min by the participant together with the clinician. This variable is measured as time of completion and number of errors made.
  (b) Digit Symbol Substitution Test (DSST): The digit symbol substitution test [23] is a neuro-physiological test sensitive to the presence of cognitive dysfunction as well as change in cognitive function across a wide range of clinical populations and is an effective method of monitoring cognitive functions over time in clinical practice. It consists of, for example, nine digit–symbol pairs (e.g., 1/−, 2/┴ … 7/Λ, 8/X, 9/=) followed by a list of digits. Under each digit, the subject should write the corresponding symbol as quickly as possible. The number of correct symbols completed within the allowed time (90 s) is recorded. Decline in symbol-copying performance is strongly correlated with age. It can be completed in approximately 2 min by the participant with the clinician. The DSST is included in the Wechsler Adult Intelligence Scale, where it is referred to as ‘Digit Symbol’ (WAIS-R) or ‘Digit-Symbol-Coding’ (WAIS-IV).

Statistical analysis:

Demographic and descriptive quantitative data were reported as the mean (standard deviation) or median (p25; p75), according to the data distribution. Qualitative variables were expressed as absolute frequencies and percentages, n (%).

To analyse the evolution of groups across the different tests, a mixed model was applied, with the follow-up visit as the within-subjects factor and groups as the between-subjects factor. When a significant interaction was detected, pairwise comparisons were performed using Tukey’s HSD correction. In cases where the effect of another variable was of interest, it was included in the model as a covariable.

Relative risks were estimated using Poisson regression with robust variance estimation. Age and sex were included as covariates in the model to assess their potential confounding effects.

Correlation analyses were conducted using Pearson’s or Spearman’s correlations, depending on the normality of quantitative variables. To assess the relationship between test outcomes and quantitative variables, the Wilcoxon–Mann–Whitney U test or median test was applied, according to the distributional characteristics of the data and the number of groups.

For comparisons involving two categorical variables, the chi-square test was performed. Normality was assessed using the Shapiro–Wilk test and visual inspections using box plots and quantile–quantile plots.

All analyses were performed in Stata V17, and a *p*-value less than 0.050 was considered statistically significant.

## 3. Results

### 3.1. Demographic Data

At the time of the last analysis review (December 2024), 714 patients had been included in the study. The distribution of patients was group A—210 patients (125 women), group B—302 patients (165 women), and group C—202 patients (91 women). One hundred and one patients had completed the follow-up visit at the time of the analyses: group A—18 patients, group B—24 patients, and group C—59 patients; the mean follow-up period was 3.6 years (SD 1.2). The demographics and medical history of each group are listed in Table 1. There were significant differences among the three groups in age, sex, educational level, plurilingualism, and work activity. Group C showed a higher percentage of males than the other groups, and the mean age was 68 years in group C, compared with 66 and 61 years in groups B and A, respectively. In group C, the percentage of individuals with a university education (28%) and bilingualism (23%) was lower. As expected, the proportion of individuals with an active working lifestyle was lower in group C (28.2%) due to retirement age.

At the follow-up visit, PTA levels were 13.2 dB in group A, 28.0 dB in group B, and 91.5 dB in group C, as shown in Table 2, indicating greater hearing loss in the treated group. The type of hearing loss per group is also shown in the table, along with other variables.

### 3.2. Hearing Loss and Postural Imbalance

**Figure 1:**
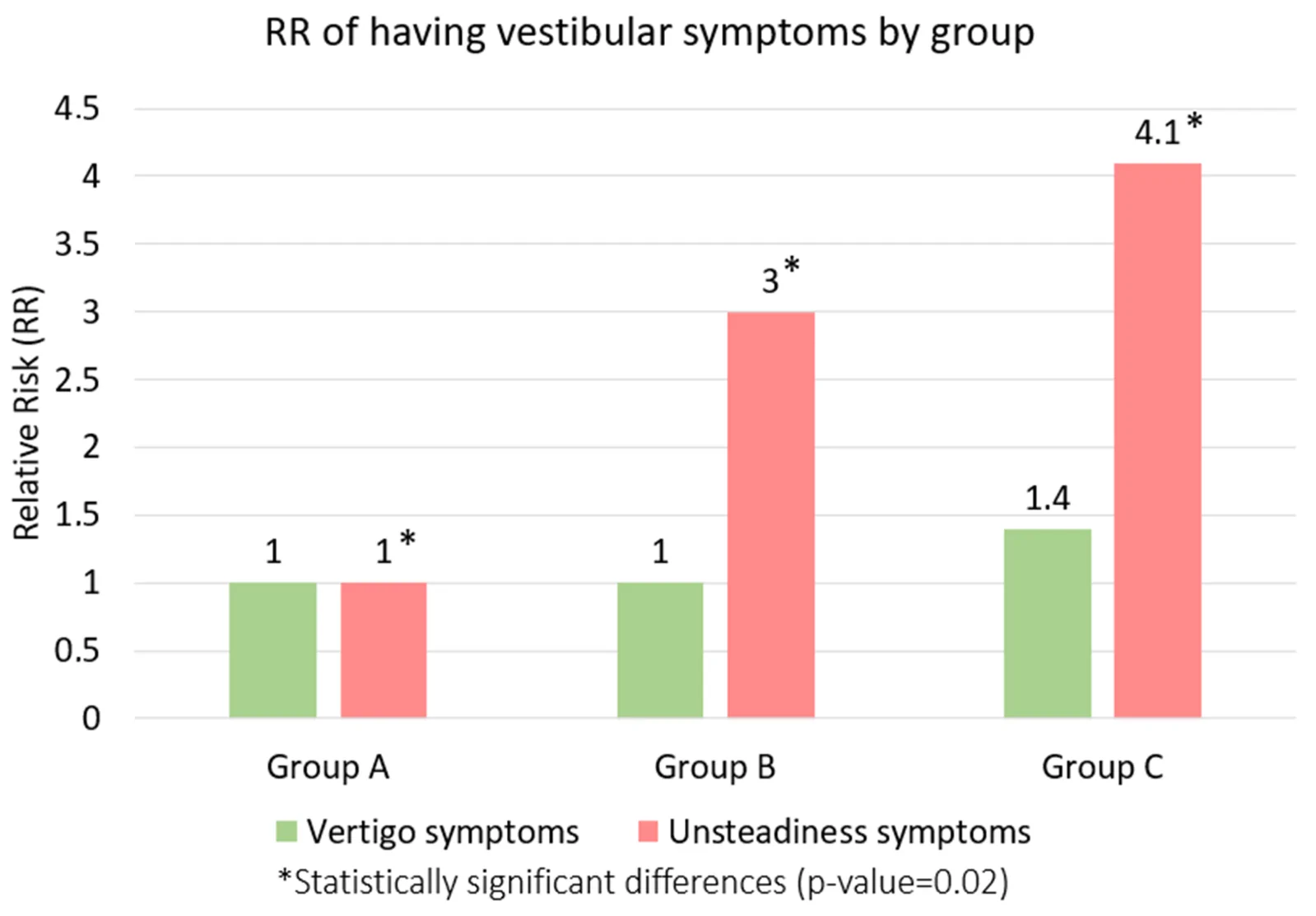
Relative risk estimates for vestibular symptoms by group.

First, we estimated the relative risk of vertigo or unsteadiness symptoms compared with the normal-hearing population (group A). Regarding vertigo, the RR of presenting symptoms was null for group B and 3.00-fold for group C, which was not statistically significant (*p* = 0.409); the RR of presenting unsteadiness symptoms was 1.4-fold for group B and 4.1-fold for group C, with statistically significant differences (*p* = 0.020). Therefore, the risk of unsteadiness increases with greater hearing loss. These results are shown in Figure 1.

Furthermore, we performed additional analyses incorporating covariates to test the robustness of our results; a model adjusting for age and sex demonstrated that neither variable was related to vertigo (*p* = 0.587 and *p* = 0.083, respectively) nor unsteadiness (*p* = 0.192 and *p* = 0.538, respectively).

When we analysed the correlation between DHI scores and hearing loss levels, the results were similar: we found a weak but statistically significant correlation, with a Spearman’s Rho of 0.199 (*p*-value < 0.001). Thus, the greater the hearing loss, the higher the DHI score.

Finally, no significant correlation was found between TUG test results and hearing loss (*p* = 0.389).

### 3.3. Hearing Loss and Cognitive Executive Function

**Figure 2:**
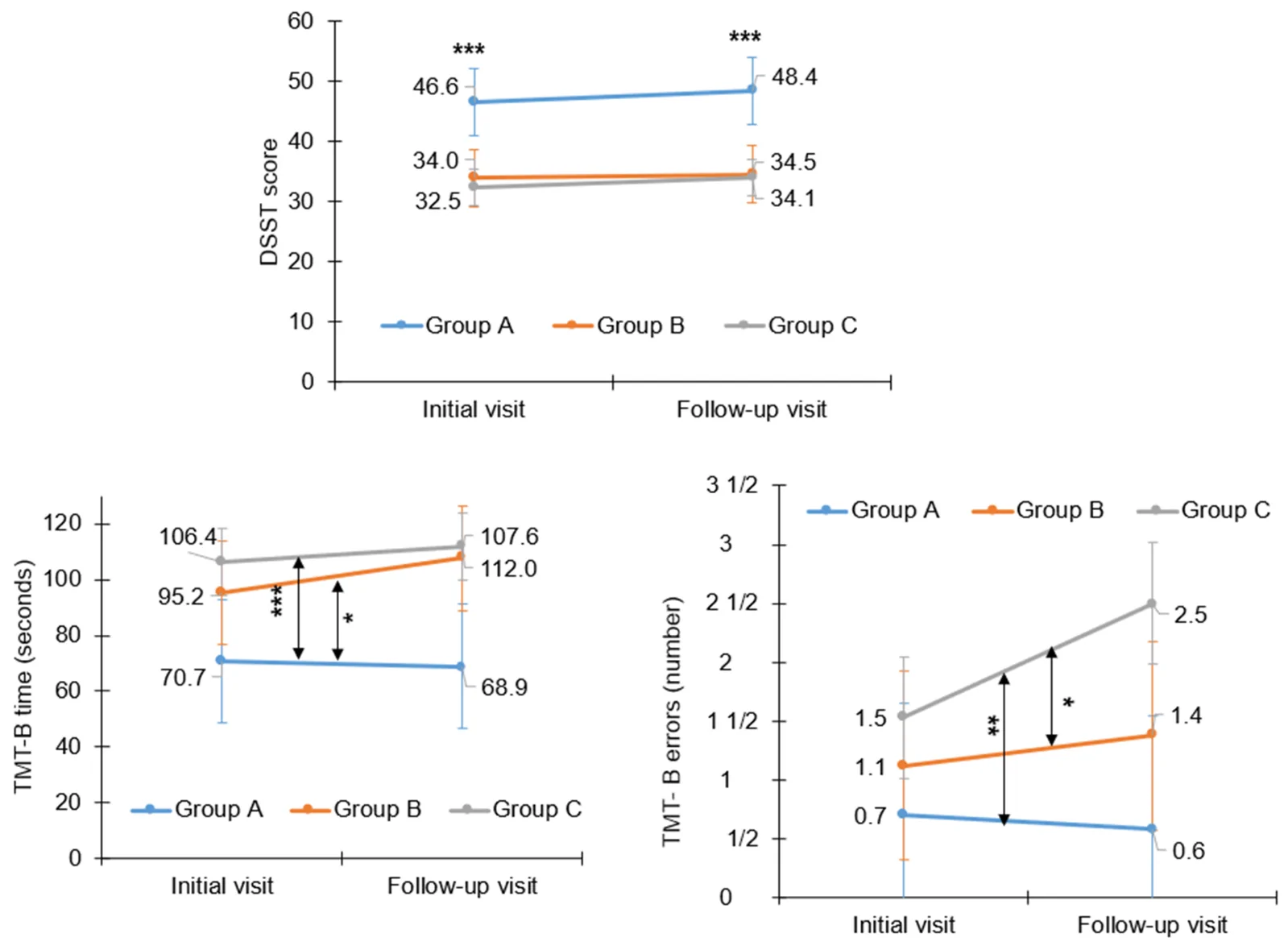
DSST scores and TMT-B (in seconds and errors) at the initial and follow-up visit according to groups. Vertical arrows indicate statistically significant between-group differences for the indicated pair of groups and asterisks denote the level of statistical significance: * *p*-value < 0.05; ** *p*-value 0.05–0.001; *** *p*-value < 0.001.

We compared executive cognitive performance across the three groups at the initial visit and at follow-up (3.6 ± 1.2 years later). First, we observed no statistically significant differences between visits, nor an interaction between visits and groups. Second, there were differences between groups in the cognitive performance tests DSST and TMT-B time (*p* < 0.001 and *p* < 0.002, respectively). For the DSST, group C differed from the other two groups with a high level of statistical significance (*p* < 0.001); for the TMT-B, group A differed significantly from group B (*p* = 0.015) and very significantly from group C (*p* < 0.001). No statistically significant differences were found between group B and group C in either DSST or TMT-B (*p* = 0.718 and *p* = 0.433, respectively). Third, for TMT-B in terms of errors, group differences were again statistically significant (*p* = 0.001). Group C differed significantly from group B (*p* = 0.032) and very significantly from group A (*p* = 0.001), whereas no differences were found between groups A and B (*p* = 0.197). These results are shown in Figure 2.

An additional analysis was conducted to adjust the correlation between PTA and cognitive tests (DSST, TMT-B time, and TMT-errors) across distinct age groups: 55–65, 66–75, 76–85, and >85 years. The results indicated statistically significant correlations (Rho < 0.3) for DSST only in the 66–75 and 76–85 age groups. Similarly, TMT-B showed a statistically significant correlation (Rho < 0.3) exclusively in the 66–75 age group. All remaining *p*-values were not statistically significant.

### 3.4. Cognitive Executive Function and Postural Imbalance

To investigate the potential relationship between cognitive executive function and vertigo or postural imbalance, a series of analyses were conducted. DSST results correlated with TUG results (Rho = −0.40; *p* < 0.001) and weakly with unsteadiness symptoms (Rho = −0.18; *p* < 0.001) but significantly with vertigo or DHI (Rho = −0.04; *p* = 0.392 and Rho = −0.04; *p* = 0.334, respectively). TMT-B completion time also correlated with TUG (Rho = 0.34; *p* < 0.001) and unsteadiness symptoms (Rho = 0.21; *p* < 0.001) and weakly with vertigo symptoms (Rho = 0.10; *p* = 0.018) and DHI (Rho = 0.11; *p* = 0.009).

**Figure 3:**
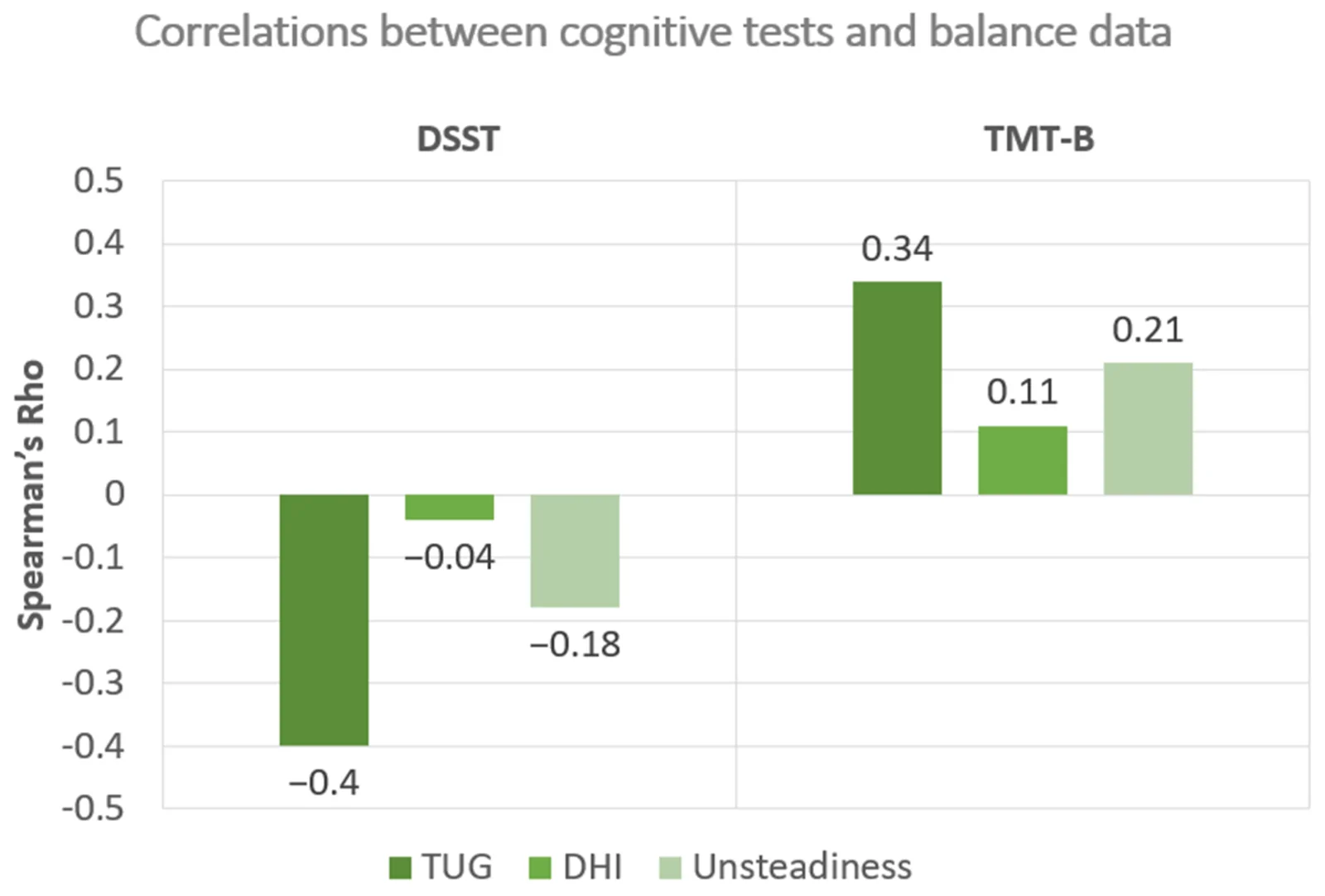
Spearman’s Rho correlations between cognitive executive function tests and balance data.

These findings suggest that cognitive executive function is not significantly associated with the DHI questionnaire or vertigo symptoms, although there is a weak correlation between cognitive performance and unsteadiness and moderate correlations with TUG results. These analyses are represented in Figure 3.

Furthermore, we observed that lower scores on these cognitive tests predicted worse performance on the TUG test, with statistically significant differences (*p* = 0.008). However, this correlation did not differ across the groups (*p* = 0.548), as groups B and C did not show significantly different progressions in TUG results in relation to cognitive performance. Conversely, there were no statistically significant differences in DHI scores between groups based on good or poor TMT-B results (*p*-value = 0.867). Further analysis of the TMT-B results, measured in both time of completion and the number of errors, revealed that patients with unsteadiness symptoms tended to have poorer performance, specifically making more errors (*p* = 0.016), but not in taking longer to complete the test (*p* = 0.062).

### 3.5. Confounding Factors

To better understand the complex connections among hearing loss, balance impairment, and cognitive executive function, an analysis was performed to identify potential confounding variables that could affect these outcomes. Specifically, we assessed whether age, sex, smoking status, cardiovascular risk factors, and educational level acted as confounders. All results are summarized in Table 3.

Regarding balance, age was found to be associated only with TUG test results (Rho = 0.372; *p* < 0.001).

The association of age and educational level with cognitive executive performance was as follows: age was significantly associated with TMT-B, specifically in the time to complete the test and also in the number of errors (Rho = −0.447; *p* < 0.001 and Rho = 0.193; *p* < 0.001; respectively), but showed no significant association with DSST (*p* = 0.717); educational level was associated with both DSST and TMT-B time of completion (*p* < 0.001 and *p* = 0.005, respectively) but not with TMT-B errors (*p* = 0.575).

Regarding hearing levels, age was not associated with PTA (*p* = 0.66), whereas PTA was associated with sex, educational level (*p* < 0.001), and the presence of cardiovascular risk factors (*p* = 0.016).

## 4. Discussion

Hearing loss is associated with both postural imbalance and cognitive executive function performance.

In terms of postural imbalance, greater hearing loss is associated with a higher likelihood of unsteadiness symptoms (RR of 1.4 in group B and 4.1 in group C compared with the normal-hearing group). Additionally, the DHI score (Rho 0.199, *p* < 0.001) increases with the degree of hearing loss. Our results suggest that postural instability may be linked to hearing loss. Nevertheless, the TUG test did not show any such relationship. The literature proposes four main explanations for the link between hearing loss and balance impairment: ageing itself, concomitant dysfunction of the cochlea and vestibular organs, the role of auditory cues in balance, and the involvement of cognitive and attentional resources in maintaining postural control [24].

Some authors have studied the patterns and processes of cochlear and vestibular ageing, suggesting that changes in these organs show both similarities and differences in pathophysiology [10]. Furthermore, functional, morphological, and molecular changes in ARVL have been studied in animal models. These findings again suggest commonalities, such as inflammation-related processes commonly upregulated in both the ageing vestibule and cochlear sensorineural substructures, as well as differences between vestibular and cochlear ageing, such as distinct genes and mechanisms that contribute separately to each process [25]. However, based on our clinical data, the strong association between hearing loss and balance impairment may be related to the theories of concomitant cochlear–vestibular dysfunction and/or the role of auditory cues in balance control.

Vestibular loss in older adults is multifactorial, though age-related decline is a key contributor, characterized by bilaterally reduced function and fewer vestibular hair cells and neurons [26,27].

In our analysis, hearing loss levels, measured by PTA, showed a significant correlation with DHI scores. Moreover, the adjusted model analyses for the covariates age and sex demonstrated that neither variable influenced the correlation between balance impairment and hearing loss. This suggests that other factors may influence this relationship, consistent with theories that attention, cognitive resources, and auditory cues—not solely the ageing process—are related to balance impairment in older adults.

Nevertheless, no correlation was found between TUG scores and hearing loss. Higher DHI scores have been found to correlate substantially with indicators of gait disturbance, including shorter strides, slower walking speeds, and greater variability in temporal gait characteristics [28]. Other authors [29] have reported that DHI scores show moderate correlations with both static and dynamic balance tests and that patients with higher DHI scores tend to have poorer balance performance and a higher incidence of falls. As highlighted by Abe-Fujisawa et al., although DHI is associated with balance performance, it may not always accurately predict fall risk in older adults [30]. Therefore, it is often recommended to use the DHI in conjunction with other balance assessments for a comprehensive evaluation.

Given these findings, hearing loss may be related to dizziness symptoms but not to mobility impairment, as reflected in the absence of correlation between hearing loss and TUG results. The shared location of the cochlea and vestibular organs within the bony labyrinth of the inner ear may explain a concomitant dysfunction, consistent with the higher RR of unsteadiness symptoms observed with greater hearing loss. Paplou et al. demonstrated common inflammation-related processes in vestibular and cochlear ageing in animal models, although distinct genes and mechanisms also contribute separately to age-related vestibular and hearing impairments [25]. The relationship between balance impairment and hearing loss is likely explained by a combined effect of some of the theories described above.

Although hearing is not traditionally considered a direct risk factor for falls, a growing body of recent research, including two systematic reviews and meta-analyses [16,18,31,32,33], has demonstrated a strong link between audiometric hearing loss and an increased incidence of falls.

In terms of cognitive executive function performance, our results indicate that hearing loss is associated with worse DSST and TMT-B scores. This difference was apparent from the early stages, as no difference was found between mild and moderate-to-severe hearing loss. Furthermore, initial treatment status did not show significant differences in the early years of follow-up, although group C was associated with a higher number of errors on the TMT-B compared to the other groups. We believe that these differences are primarily attributable to the hearing impairment itself rather than to hearing aid use, reflected by the greater severity of hearing loss in group C. Nonetheless, hearing loss clearly shows an association with cognitive abilities. The additional analysis, adjusting the correlation between PTA and cognitive tests (DSST, TMT-B) by age group, showed no significant correlations across all age groups, only in two age groups for DSST and one for TMT-B time. This suggests that age does not modify the association between these variables, consolidating our other findings; or, if it does at some point, it is not the only contributing factor. Thus, although both hearing and cognition decline with age, the stability of this association over time suggests that other factors are also involved.

In recent years, many studies have investigated the association between hearing loss and cognitive decline; according to the literature, hearing loss is associated with an increased risk of cognitive decline or dementia [6,34,35,36,37]. In our previous observational study (n = 714), a negative correlation was found between the DSST cognitive test and the duration of hearing loss prior to treatment (−0.26; *p* = 0.003), suggesting that delayed treatment predisposes patients to cognitive decline [38]. Another study demonstrated that peripheral hearing impairment is independently associated with accelerated brain atrophy, both globally and in regional volumes concentrated in the right temporal lobe [39]. A recent study even demonstrated a meaningful clinical improvement in cognitive function after cochlear implantation in older adults with severe hearing loss at risk of mild cognitive impairment [40].

It remains unknown whether ARHL leads to cognitive decline and thus higher fall risk or whether ARHL and balance impairment share a physiopathological mechanism that subsequently leads to cognitive decline. The association between hearing loss and falls may be mediated by cognitive load and reduced attentional resources, which are critical for postural control; decrements in these resources due to hearing loss may impair postural balance in real-world situations and increase fall risk [34]. These two pathways are of particular interest given that hearing loss is highly prevalent yet substantially undertreated in older adults [5].

Regarding the relationship between balance performance and cognitive function, we observed higher TUG test scores when DSST and TMT-B results were worse, suggesting that cognitive executive function impairment may affect functional capacity, mobility, or balance. In contrast, no statistically significant correlation was found between the presence of vestibular symptoms or the self-perceived impact of dizziness (DHI) and either cognitive test. Only a weak negative correlation (Rho < 0.3) was found between both cognitive tests and the presence of unsteadiness symptoms. In general terms, patients with worse TMT-B results also had worse TUG scores (*p* = 0.037) than those with better TMT-B performance, but there were no differences in DHI scores according to TMT-B results (*p* = 0.876).

Considering these results together, we suggest that cognitive executive function is not associated with balance impairment in terms of vestibular symptoms or self-perceived impact of dizziness. However, we demonstrated that patients with cognitive impairment exhibit worse agility and functional capacity as reflected in TUG test results, consistent with the prior literature [41,42]. Thus, we hypothesise that patients with cognitive executive function impairment are not subjectively more unstable but are instead “slower in movement” compared to those without cognitive decline, and this finding is independent of hearing loss severity.

In contrast, several studies have demonstrated a significant relationship between balance and cognition in older adults [43,44,45,46,47]. Rather than establishing that cognitive decline leads to balance impairment, as initially thought, some of these studies suggest that balance symptoms may be an early marker of cognitive decline. Furthermore, some even suggest that this may be useful in screening individuals at risk of developing dementia [43,44,45,47].

A systematic review and meta-analysis highlighted that evidence regarding the relationship between cognition and balance remains inconclusive, suggesting that the type of balance task influences this relationship; however, our results suggest that a relationship does exist between balance, as measured by TUG, and cognitive executive performance. These findings have implications for assessment, treatment planning, fall prevention, functional training, cognitive–motor integration, and rehabilitation outcomes [48].

Finally, we sought to identify potential confounding variables affecting these three relationships. As expected, age and educational level were associated with TMT-B results, consistent with the previous literature [49]. However, while educational level was also associated with DSST performance, age was not. This finding is somewhat unexpected given the literature, as age is commonly reported to correlate with DSST scores [50]. Consequently, these specific results will be re-evaluated in the longitudinal follow-up and subsequent analyses of this patient cohort.

Regarding the balance assessment tools used (DHI and TUG), only age was associated with TUG results, which is expected given that age affects movement agility and functional capacity, as reported in the literature [41,50].

Sex was correlated with hearing loss levels, but, surprisingly, age did not show a statistically significant association, in contrast to our previous findings, which showed a correlation between age and hearing level at 4 kHz [38]. These results should be interpreted with caution, as this is part of a longitudinal study with incomplete follow-up, potentially introducing bias. We identified that cardiovascular risk factors were associated with hearing loss levels, consistent with descriptions that inflammation from factors like smoking, hypertension, and diabetes contributes to this decline [51]. Conditions such as cardiovascular disease, diabetes mellitus, and Alzheimer’s disease all involve inflammation and show an association with ARHL [52]. In our cohort, smoking did not show this reported association. Lastly, educational level appears to affect hearing loss, a relationship that requires further research to explore potential explanations, such as occupational noise exposure and/or living environment (rural vs. urban), among other variables.

In general, age was observed to be associated with balance and cognition. However, our adjusted analyses incorporating age and sex revealed that age did not significantly alter the statistical correlations or relative risk estimations described before. Therefore, while age undoubtedly correlates with the individual variables, consistent with the existing literature, it is not the only factor at play in how they relate to one another.

These results could be instrumental in identifying comprehensive risk factors for balance impairment in the elderly, whether associated with hearing loss or cognitive decline.

Future research should address whether early treatment of hearing loss can mitigate its non-auditory consequences, such as cognitive decline, balance impairment, and reduced quality of life, among others. Longitudinal and interventional studies are needed to determine the extent to which hearing rehabilitation functions not only as tertiary prevention but also as secondary prevention by limiting the progression of these downstream effects. Clarifying these relationships would help refine public health strategies and prioritize early auditory intervention as a potential modifier of broader health outcomes.

## 5. Limitations

First, this prospective observational study is subject to limitations related to its ongoing nature. The low proportion of participants with completed follow-up at the time of analysis reflects that most individuals are still pending scheduled visits rather than being lost. However, the high and differential attrition across exposure groups may introduce selection bias if completion of follow-up is associated with both exposure and outcome. Consequently, current estimates may be imprecise and subject to informative censoring. These analyses will be updated as additional follow-up data accrue.

Another limitation is the absence of objective vestibular status assessment using vestibular function tests. While applying tests such as vHIT and posturography, or the Sensory Organization Test subtest, to a large sample is challenging, we prioritized the Timed Up and Go (TUG) test. TUG is a reliable tool for evaluating mobility, balance, walking pattern, and fall risk, which we deemed more relevant to our study’s aim than a test like vHIT, where a normal result does not exclude the possibility of a balance disorder.

A further possible limitation of this study is the absence of information on participants’ physical activity levels.

Another limitation of this study is the substantial difference in the PTA between the untreated group (B) and the treated group (C). However, this may represent usual clinical practice and patient-related factors and could also be interpreted as evidence that differences begin to emerge across all degrees of hearing loss, including early stages.

Finally, a potential selection bias exists, as participants were volunteers, who are typically more health-conscious, physically active, and attentive to their diet and overall well-being, which may limit the generalizability of the findings.

## 6. Conclusions

Postural imbalance and cognitive executive function performance are related to hearing loss. However, there is no evidence linking dizziness to cognitive executive function, although an association with TUG results was found. Therefore, hearing loss is correlated with balance and cognition, while cognition itself is also related to postural balance.

## Figures and Tables

**Table 1:** Demographic data at the recruitment visit. Results are presented as n (%) or mean (±SD).

Characteristics	Group A (n = 210)	Group B (n = 302)	Group C (n = 202)	*p*-Value	Total n
**Sex(Male/** **Female)**	126 (60%) Female	166 (55%) Female	91 (45%) Female	*p* = 0.011	383 (53.6%) Female
**Age**	61.8 (±5.9) Years	66.6 (±8.1) Years	68.3 (±8.7) Years	*p* < 0.001	65.7 (±8.1) Years
**Habitat** **(Rural/Urban)**	174 (83%) Urban	236 (78%) Urban	152 (75%) Urban	NSD	562 (78.7%) Urban
**Assistance (None/Family)**	208 (99%) None	293 (97%) None	200 (99%) None	NSD	701 (98.2%) None
**Education** **level**	84 (40%) University or More	115 (38%) University or More	59 (29%) University or More	*p* = 0.044	258 (36.1%) University or More
**Languages (Mono/Plurilingual)**	136 (65%) Monolingual	211 (70%) Monolingual	156 (77%) Monolingual	*p* = 0.027	503 (70.4%) Monolingual
**Work activity**	111 (53%) Yes	106 (35%) Yes	57 (28%) Yes	*p* < 0.001	274 (38.4%) Yes
**Tobacco**	116 (55%) No 90 (38%) Ex-smoker	142 (47%) No 115 (43%) Ex-smoker	91 (45%) No 91 (45%) Ex-smoker	NSD	349 (48.9%) No 296 (41.5%) Ex-smoker
**Cardiovascular** **disease**	59 (28%) Yes	94 (31%) Yes	63 (31%) Yes	NSD	216 (30.3%) Yes
**Psychiatric disease**	38 (18%) Yes	63 (21%) Yes	34 (17%) Yes	NSD	135 (18.9%) Yes
**Neurological** **disease**	8 (4%) Yes	24 (8%) Yes	16 (8%) Yes	NSD	48 (6.7%) Yes
**Endocrine disease**	65 (31%) Yes	94 (31%) Yes	65 (32%) Yes	NSD	224 (31.4%) Yes
**Mobility** **(Normal/Assisted)**	2 (1%) Assisted	6 (2%) Assisted	10 (5%) Assisted	NSD	18 (2.5%) Assisted

**Table 2:** Baseline characteristics of the study population by group at the follow-up visit. Results are presented as n (%) or mean (SD) when applicable or median (p25; p75) if the variable did not follow a normal distribution.

Results at Follow-Up (Mean 3.6 + 1.2 Years)	Group A (n = 18)	Group B (n = 24)	Group C (n = 59)
**Hearing loss median by group (dB)**	13.2 (10.0; 17.5)	28.0 (24.0; 32.7)	91.5 (64.0; 103.0)
**Hearing loss type by group**	N/A	Conductive: 0 Sensorineural: 22 (91.7%) Mixed: 2 (8.3%)	Conductive: 3 (5.1%) Sensorineural: 50 (84.7%) Mixed: 6 (10.2%)
**Hearing device used**	N/A	N/A	HA-none: 3 (5.1%) HA-HA: 19 (32.2%) HA-CI: 22 (37.3%) ICVO-HA: 1 (1.7%) IC-none: 11 (18.6%) IC-IC: 1 (1.7%) IC-ICVO: 1 (1.7%) IAOM-ICVO: 1 (1.7%)
**DSST score by group**	48.4 (9.3)	34.5 (15.4)	34.1 (13.4)
**TMT-B by group (seconds/errors)**	68.9 s (56.5; 85.5)/0.6 e (0; 1)	112.0 s (64.0; 125.0)/ 1.4 e (0; 2)	107.6 s (68.0; 120.0)/ 2.5 e (0; 3)
**TUG test in seconds**	8 s (7; 9)	9 s (8; 10)	9 s (8; 12)
**Unsteadiness symptoms (% yes, % no)**	Yes: 2 (11.1%) No: 16 (88.9%)	Yes: 7 (29.2%) No: 17 (70.8%)	Yes: 25 (42.4%) No: 34 (57.4%)
**DHI median [and score mean] by group**	0 and [2]	0 and [4, 88]	0 and [8, 35]

**Table 3:** Summary of the correlation analysis results identifying confounding factors potentially associated with hearing loss, balance impairment, or cognitive decline. Coloured cells show statistically significant relationships.

Variables Correlation	Hearing Loss	Postural Balance		Cognitive Performance		
	PTA	DHI Score	TUG Test	DSST	TMT-B Time	TMT-B Errors
**Age**	Rho = 0.092 *p* = 0.066	Rho = −0.046 *p* = 0.355	Rho = 0.372 *p* < 0.001	Rho = 0.025 *p* = 0.717	Rho = −0.447 *p* < 0.001	Rho = 0.193 *p* < 0.001
**Sex (M/F)**	*p* < 0.001	*p* = 0.612	*p* = 0.707	*p* = 0.284	*p* = 0.158	*p* = 0.825
**Cardiovascular risk factors**	*p* = 0.016	*p* = 0.974	*p* = 0.395	*p* = 0.188	*p* = 0.524	*p* = 0.803
**Smoking (yes/no)**	*p* = 0.694	*p* = 0.534	*p* = 0.834	*p* = 0.647	*p* = 0.913	*p* = 0.825
**Education level**	*p* < 0.001	*p* = 0.536	*p* = 0.205	*p* < 0.001	*p* = 0.005	*p* = 0.575

## Data Availability

The raw data supporting the conclusions of this article will be made available by the authors upon request.

## Abbreviations

The following abbreviations are used in this manuscript:

HL	Hearing loss
ARHL	Age-related hearing loss
dBHL	Decibel hearing levels
DHI	Dizziness Handicap Index
TUG	Timed Up and Go
TMT-B	Trail Making Test Part B
RR	Relative risk estimation
DSST	Digit Symbol Substitution Test
PTA	Pure-tone average
